# Inositol hexakisphosphate kinase 1 (IP6K1) activity is required for cytoplasmic dynein-driven transport

**DOI:** 10.1042/BCJ20160610

**Published:** 2016-09-27

**Authors:** Manasa Chanduri, Ashim Rai, Aushaq Bashir Malla, Mingxuan Wu, Dorothea Fiedler, Roop Mallik, Rashna Bhandari

**Affiliations:** 1 Laboratory of Cell Signalling, Centre for DNA Fingerprinting and Diagnostics, Nampally, Hyderabad 500001, India; 2 Graduate Studies, Manipal University, Manipal, India; 3 Department of Biological Sciences, Tata Institute of Fundamental Research, Homi Bhabha Road, Mumbai 400005, India; 4 Department of Chemistry, Princeton University, Washington Rd, Princeton, NJ 08544, U.S.A.

**Keywords:** dynactin, dynein, inositol hexakisphosphate kinase 1, inositol pyrophosphates, protein pyrophosphorylation

## Abstract

Inositol pyrophosphates, such as diphosphoinositol pentakisphosphate (IP_7_), are conserved eukaryotic signaling molecules that possess pyrophosphate and monophosphate moieties. Generated predominantly by inositol hexakisphosphate kinases (IP6Ks), inositol pyrophosphates can modulate protein function by posttranslational serine pyrophosphorylation. Here, we report inositol pyrophosphates as novel regulators of cytoplasmic dynein-driven vesicle transport. Mammalian cells lacking IP6K1 display defects in dynein-dependent trafficking pathways, including endosomal sorting, vesicle movement, and Golgi maintenance. Expression of catalytically active but not inactive IP6K1 reverses these defects, suggesting a role for inositol pyrophosphates in these processes. Endosomes derived from slime mold lacking inositol pyrophosphates also display reduced dynein-directed microtubule transport. We demonstrate that Ser51 in the dynein intermediate chain (IC) is a target for pyrophosphorylation by IP_7_, and this modification promotes the interaction of the IC N-terminus with the p150*^Glued^* subunit of dynactin. IC–p150*^Glued^* interaction is decreased, and IC recruitment to membranes is reduced in cells lacking IP6K1. Our study provides the first evidence for the involvement of IP6Ks in dynein function and proposes that inositol pyrophosphate-mediated pyrophosphorylation may act as a regulatory signal to enhance dynein-driven transport.

## Introduction

Inositol pyrophosphates are small-molecule second messengers composed of an inositol ring containing pyrophosphate groups in addition to monophosphates [1,2]. They occur ubiquitously in all eukaryotes and participate in many cellular processes, including DNA repair, stress response, apoptosis, phosphate metabolism, and energy homeostasis [3]. 5-Diphosphoinositol pentakisphosphate (5-IP_7_) is the most abundant inositol pyrophosphate in mammalian cells with cellular concentrations ranging from 0.5 to 3 µM [4]. 5-IP_7_ is synthesized from inositol hexakisphosphate (IP_6_) by inositol hexakisphosphate kinases (IP6Ks), which have three homologs in mammals, such as IP6K1, IP6K2, and IP6K3 [5–7]. Other inositol pyrophosphates, such as 1-diphosphoinositol pentakisphosphate (1-IP_7_) and 1,5-bis-diphosphoinositol tetrakisphosphate (IP_8_), are generated by another class of enzymes called PP-IP5 kinases [7] and occur in lower amounts in most eukaryotic cells [4]. Inositol pyrophosphates can modulate protein function in two ways: (a) by direct binding to a target protein or (b) by conferring a posttranslational modification known as pyrophosphorylation [8,9]. The latter mechanism involves the transfer of a high-energy β-phosphate from an inositol pyrophosphate such as 5-IP_7_ to a phosphorylated serine residue to form pyrophosphoserine and has been shown to regulate protein–protein interactions [10,11].

In addition to their extensive role in regulating metabolic and signaling pathways, inositol pyrophosphates have been shown to participate in different vesicle trafficking processes. In yeast, vesicle transport takes place along the actin cytoskeleton with the help of myosin motors [12]. In mammals, short-range movement of endocytic and exocytotic vesicles near the plasma membrane is also actin–myosin dependent [12]. Long-range transport in mammalian cells occurs on the microtubule cytoskeleton and is driven by two classes of motor proteins. These are kinesins, which move vesicles towards the plus-end of microtubules extending to the cell periphery and cytoplasmic dynein, which carries vesicles towards the minus-end near the nucleus [13]. Inositol pyrophosphates have been shown to negatively regulate the interaction of the kinesin motor Kif3A with the adaptor protein 3 (AP3) [10]. 5-IP_7_-mediated pyrophosphorylation of the AP3 β-subunit reduces its interaction with Kif3A and suppresses the release of viral particles from cells. Budding yeast lacking inositol pyrophosphates shows a fragmented vacuolar morphology due to defective endosomal sorting [14,15]. Inositol pyrophosphates promote insulin exocytosis from pancreatic β-cells by increasing the readily releasable pool of insulin-containing vesicles and enhancing insulin release from these vesicles [16]. While the molecular basis for the observations made in yeast and pancreatic β-cells remains elusive, these studies suggest a role for inositol pyrophosphates in actin–myosin and microtubule-dependent kinesin-driven processes. Till date, however, there have been no studies investigating the role, if any, played by inositol pyrophosphates in dynein-driven vesicular transport.

In the present study, we observe impaired dynein-dependent transport in mammalian cells lacking IP6K1 and in slime mold lacking the IP6K homolog, I6KA. The dynein intermediate chain (IC), a noncatalytic dynein subunit, undergoes IP_7_-mediated pyrophosphorylation on its N-terminus, which enhances its binding to the p150*^Glued^* subunit of dynactin. The loss of IP6K1 leads to reduced interaction of IC with p150*^Glued^* and decreased dynein recruitment to cellular membranes. Thus, our findings uncover a previously unknown role for inositol pyrophosphates and protein pyrophosphorylation in dynein-driven vesicle transport.

## Materials and methods

### Cell lines and expression constructs

All mouse and human cell lines were grown in Dulbecco's modified Eagle's medium (DMEM, Life Technologies) supplemented with 10% fetal bovine serum (Life Technologies), 1 mM l-glutamine (Life Technologies), 100 U/ml penicillin, and 100 µg/ml streptomycin (Life Technologies). The generation of single cell-derived *Ip6k1*^+/+^ and *Ip6k1*^−/−^ mouse embryonic fibroblasts (MEFs) and *Ip6k1*^−/−^ MEFs expressing catalytically active or inactive forms of IP6K1 has been described earlier [17]. Briefly, retrovirus particles carrying pCX4Neo plasmid containing the coding DNA sequence for IP6K1 (GenBank Accession number AF177144) or an inactive form of IP6K1 (nucleotide changes A676G, A677C, and T1000G, resulting in mutations Lys226Ala and Ser334Ala) were used to transduce *Ip6k1*^−/−^ MEFs. Single cell-derived lines stably expressing active or inactive IP6K1 were cultured in medium supplemented with G418 (200 µg/ml, Sigma-Aldrich). Wild-type and *i6kA*^−^ (AX2 strain background) *Dictyostelium discoideum* described recently [18] were obtained from DictyBase (http://dictybase.org) and grown in HL-5 medium (14 g peptone, 7 g yeast extract, 13.5 g glucose, 0.5 g KH_2_PO_4_ and 0.5 g Na_2_HPO_4_ dissolved in 1 l of water, pH 6.5) containing 200 U/ml penicillin and 200 µg/ml streptomycin. Full-length mouse dynein IC-2C, plasmid p199 Dync1i2.C (Ex1a) [19], was a gift from Elizabeth Fisher (Department of Neurodegenerative Disease, UCL Institute of Neurology, London, United Kingdom) (Addgene plasmid # 26449; GenBank Accession number NM_010064). This was used as a template to obtain cDNA encoding IC-2C fragments corresponding to amino acid residues 1–70 and 1–111, which were subcloned into the BamHI and NotI restriction enzyme sites in the plasmid pGEX-6P-1 (GE Life Sciences) or pCDNA 3.1(+) (Invitrogen). The IC(1–111)S51A mutants were generated from pGEX-6P-1-IC(1–111) or pCDNA-IC(1–111) using the QuikChange II Site-Directed Mutagenesis Kit (Agilent) as per the manufacturer's instructions.

### Mice

Animal studies were carried out as per guidelines provided by the Committee for the Purpose of Control and Supervision of Experiments on Animals, Ministry of Environment, Forest, and Climate Change, Government of India. Mice were housed in the Centre for DNA Fingerprinting and Diagnostics animal facility located within the premises of Vimta Labs, Hyderabad. All animal experiments were approved by the Institutional Animal Ethics Committee (Protocol number PCD/CDFD/02 — version 2). The *Ip6k1* gene knockout mouse was generated as previously described [20] and backcrossed for seven generations into the C57BL/6 strain. *Ip6k1* heterozygous mice were bred to generate littermate *Ip6k1^+/+^* and *Ip6k1*^−/−^ mice. Eight- to 20-week-old age-matched female mice were used to generate peritoneal macrophages as per standard procedures. Briefly, mice were injected (i.p. 50 µl/g body weight) with 3% (w/v) thioglycollate broth (Sigma-Aldrich). Four days later, mice were killed by CO_2_ inhalation, and peritoneal macrophages were isolated by flushing the peritoneal cavity with 10 ml of Dulbecco's phosphate-buffered saline (PBS, Life Technologies). Macrophages were maintained for 24–48 h in the same medium as MEFs prior to experiments.

### Reagents and antibodies

Fluorescent ligands Alexa Fluor 488 transferrin (Alexa488 Tfn, T-13342) and Alexa Fluor 594 cholera toxin B subunit (CT-B; C-34777) were purchased from Molecular Probes (Life Technologies). Primary antibodies and their respective dilutions used for immunofluorescence (IF) and immunoblotting (IB) were: anti-EEA1 (ab2900, Abcam; 1:100 IF), anti-GM130 (610822, BD Biosciences; 1:200 IF, 1:2000 IB), anti-α-tubulin (T9026, Sigma-Aldrich; 1:5000 IB), anti-p150*^Glued^* (ab11806, Abcam; 1:2500 IB), anti-GST antibody (ab19256, Abcam; 1:20 000 IB), and anti-dynein IC antibodies (MAB1618, Millipore and D5167, Sigma-Aldrich; 1:000 IB). All fluorochrome-conjugated secondary antibodies were obtained from Molecular Probes (Life Technologies).

### Immunofluorescence

Cells grown on coverslips were fixed with 4% paraformaldehyde (PFA), permeabilized in 0.1% Triton-X 100 for 5 min, and incubated in blocking solution (2% BSA in PBS) for 1 h at room temperature. Cells were then incubated for 2–18 h in primary antibodies diluted appropriately in blocking solution, followed by incubation with secondary antibodies diluted in blocking solution for 1 h. Coverslips were mounted on glass slides using mounting medium containing DAPI (H-1200, Vector Labs). Images were acquired using an LSM 510 (LSM acquisition software) or LSM 700 (Zen acquisition software) confocal microscope (Zeiss) equipped with 405, 488, and 555/561 nm lasers and fitted with a ×63, 1.4 N.A. objective.

### Fluorescent ligand uptake and trafficking assays

Tfn endocytosis and trafficking assays were done as previously described [21] with slight modifications. To monitor Tfn endocytosis by flow cytometry, MEFs grown in 35 mm dishes were serum-starved for 30 min in 0.5% BSA-containing DMEM, followed by 5 min incubation with 25 µg/ml Alexa488 Tfn at 37°C. Cells were washed with cold PBS, trypsinized, and transferred to chilled tubes containing DMEM. The cells were pelleted by centrifugation and resuspended in 3% PFA. At least 10 000 cells were analyzed by flow cytometry (BD Accuri C6) using a 488 nm laser. For microscopy, MEFs grown on glass coverslips were incubated in serum-free medium for 1 h at 37°C, followed by 25 µg/ml Alexa488 Tfn on ice for 30 min. Cells were allowed to take up the bound Alexa488 Tfn for 5 min at 37°C to monitor endocytosis or for 1 h at 37°C to monitor accumulation in the endocytic recycling compartment (ERC). Cells were washed with chilled Dulbecco's PBS (Life Technologies), fixed using 4% PFA, and, where required, subjected to IF with EEA1 antibody. To measure CT-B binding to the plasma membrane, MEFs were serum-starved for 1 h at 37°C and then incubated with 5 µg/ml Alexa Fluor 594 CT-B for 1 h on ice. Cells were washed with Dulbecco's PBS and fixed with 4% PFA. Coverslips were mounted in mounting medium containing DAPI (H-1200, Vector Labs), and images were acquired as above.

### Image analysis

Where indicated, images were subjected to adjustment of tonal range on the whole image using Adobe Photoshop (levels adjustment) to improve visualization for representation purposes. Such adjustments were identical for all images in a single assay. For quantification, raw images were analyzed. To assess Tfn uptake and CT-B binding, fluorescence intensity per cell was quantified using the ImageJ ‘measure’ tool. Individual cells were marked using the ‘selection tool’ and the integrated density value of the fluorescence signal in each cell was recorded. Cells with accumulation of Tfn in ERC-like structures were scored as described previously [21]. Analysis of Golgi morphology was performed as described earlier [22]. Colocalization analysis was performed in ZEN 2012 SP1 software (Zeiss). Briefly, a fixed threshold was set across all images to exclude background fluorescence and the percentage of colocalization of EEA1 with Tfn in the entire cell was calculated by the ratio of the number of colocalized pixels of EEA1 to the total EEA1 pixels.

### Live cell imaging

MEFs grown on glass-bottom dishes were serum-starved for 1 h and incubated with 5 µg/ml Alexa Fluor 594 CT-B for 30 min at 37°C in serum-free medium. After adding complete medium, cells were placed in an incubation chamber with controlled temperature (37°C) and CO_2_ (5%) on an inverted Zeiss LSM510 confocal microscope. Images were acquired at intervals of 10 s for 200 s using the 561 nm laser with a ×63 1.4 N.A. objective. CT-B vesicles were tracked over several frames using the ImageJ ‘manual tracking’ plugin. Only vesicles that were distant from the nucleus and plasma membrane were tracked.

### Phagosome distribution assay

Peritoneal macrophages derived from *Ip6k1*^+/+^ and *Ip6k1*^−/−^ mice grown on glass-bottom dishes were serum-starved for 1 h and incubated with 750 nm carboxylated latex beads (07759, Polysciences) for 1 h to allow phagocytosis. The unphagocytosed beads were washed with serum-containing media, and the cells were incubated for 1 h in the same medium at 37°C. Differential interference contrast (DIC) images were acquired using a live cell multipoint imaging system (Nikon Ti Eclipse) equipped with a ×100 1.4 N.A. objective, ×1.5 intermediate magnification, and a CoolSnap HQ CCD camera. Images were analyzed by ImageJ to identify the cell and nuclear boundaries and to examine the movement of phagocytosed beads along the long edge of the cell towards the nucleus. The fractional distance of each bead from the nuclear centroid was calculated as the ratio of the distance of the bead from the center of the nucleus to the distance of the cell membrane from the center of the nucleus along the line joining the centroid and bead.

### Endosome motility analysis in *D. discoideum*

Endosome motility in *D. discoideum* was performed essentially as described earlier [23]. Briefly, 4–6 × 10^8^ cells of wild-type and *i6kA*^−^ amoebae were harvested, washed with Sorenson's phosphate buffer (pH 6.0), resuspended in 1:1 (w/v) lysis buffer [30 mM Tris–HCl, pH 8.0, 4 mM EGTA, 3 mM DTT, 5 mM benzamidine HCl, 5 mM PMSF containing 30% (w/v) sucrose and protease inhibitor cocktail (Roche)], and lysed by passing through a polycarbonate filter with 5 µm pore size. The crude lysate was centrifuged at 2000 ***g*** for 5 min at 4°C to obtain the postnuclear supernatant (PNS). PNS (0.5 µl) was diluted with 18.5 µl of lysis buffer and 1 µl of ATP regeneration system (1 mM ATP, 1 mM MgCl_2_, 2 mM creatine phosphate, and 2 U/ml creatine kinase). This motility mixture was passed into a flow cell containing polarity-labeled microtubules. Optical trap experiments were performed to assess the direction of motility of refractile endosomes by customized video-enhanced DIC microscopy (Nikon) using a ×100 oil immersion objective. Motion of single endosomes was recorded at 30 frames/s and tracked offline with ∼5 nm resolution, followed by analysis of motion using Bayesian optimization to extract velocities of motion [24].

### Protein purification, phosphorylation, and pyrophosphorylation

GST-tagged dynein IC fragments corresponding to residues 1–70 and 1–111 were expressed in *Escherichia coli* BL21(DE3) strain and purified using glutathione-agarose beads (GE Life Sciences) by standard procedures. Radiolabeled IP_7_ synthesis, CK2-mediated phosphorylation, and IP_7_-mediated pyrophosphorylation were performed as described earlier [9]. For pyrophosphorylation assays, beads were first treated with CK2 enzyme (New England Biolabs) in protein kinase buffer (New England Biolabs) and 0.5 mM Mg^2+^-ATP for 30 min at 30°C, then washed with cold PBS, resuspended in IP_7_ pyrophosphorylation buffer (25 mM HEPES, pH 7.4, 50 mM NaCl, 6 mM MgCl_2_, and 1 mM DTT) containing 5–7 µCi 5[β-^32^P]IP_7_, and incubated at 37°C for 15 min. LDS sample buffer (NP0008, Life Technologies) was added to the beads, and the sample was heated at 95°C for 5 min. Proteins were resolved on a 4–12% NuPAGE Bis–Tris gel (Thermo Fisher Scientific), transferred to a PVDF membrane (GE Life Sciences), and pyrophosphorylation was detected using a phosphorimager (Typhoon FLA-9500). The amount of protein loaded was quantified based on the intensity of Ponceau S staining, which has been shown to be linear with increasing protein amount up to 140 µg [25]. Radiolabeled protein as a fraction of total protein was quantified using ImageJ.

For back-phosphorylation assays, dynein IC was immunoprecipitated from *Ip6k1*^+/+^ and *Ip6k1*^−/−^ MEFs. Protein on beads was subjected to CK2-mediated phosphorylation by incubating with CK2 enzyme (New England Biolabs) in protein kinase buffer (New England Biolabs) in the presence of 0.5 mM Mg^2+^-ATP and 1–2.5 µCi [γ^32^-P]ATP for 30 min at 30°C. For back-pyrophosphorylation assays, beads were incubated with 5[β-^32^P]IP_7_ as above, but without CK2 pre-phosphorylation. Radiolabeled proteins were detected using a phosphorimager (Typhoon FLA-9500), and total protein was subsequently detected by IB. Care was taken to ensure that the chemiluminescence signal was below saturation level. Radiolabeled protein as a fraction of total immunoprecipitated protein was quantified using ImageJ. We observed a variation in the apparent molecular weight of dynein IC in NuPAGE Bis–Tris gels compared with SDS–PAGE Tris-Glycine gels.

### Mass spectrometry identification of phosphosites

GST-tagged IC-2C fragments were purified from *E. coli*, phosphorylated by CK2 as described above but without the incorporation of radiolabeled ATP, and resolved on a 4–12% Nu-PAGE Bis–Tris gel. Bands were visualized with Simply Blue Safe Stain (Invitrogen), excised, and sent to the Taplin Biological Mass Spectrometry Facility (TMSF), Harvard Medical School, Boston, USA for phosphosite identification. Briefly, the gel pieces were diced and subjected to a modified in-gel trypsin digestion and peptide extraction [26]. Samples were loaded via an FAMOS autosampler (LC Packings) onto a packed C18 reverse-phase HPLC capillary column (100 µm inner diameter × ∼30 cm length) and resolved with an acetonitrile gradient (2.5–97.5% acetonitrile and 0.1% formic acid). Each eluted peptide was subjected to electrospray ionization and entered into an LTQ Orbitrap Velos Pro ion-trap mass spectrometer (Thermo Fisher Scientific). Detection, isolation, and fragmentation of eluted peptides were conducted to generate a tandem mass spectrum of fragment ions specific for each peptide. The acquired fragmentation pattern was matched to translated nucleotide or protein databases using SEQUEST (Thermo Finnigan) to determine the corresponding peptide sequence. The modification of 79.9663 mass units to Ser, Thr, and Tyr was included in the database searches to identify phosphopeptides. Phosphorylation assignments were determined by the Ascore algorithm [27]. According to instructions from TMSF, a phosphopeptide with an Ascore of >19 was considered to be phosphorylated with 99% certainty.

### Protein interaction studies

To monitor interaction of the dynein IC N-terminus with endogenous p150*^Glued^*, 5 µg of GST-tagged dynein IC(1–111) bound to glutathione-agarose beads was phosphorylated with CK2 and unlabeled Mg^2+^-ATP, and incubated with 50 µM 5-IP_7_, 5-PCP-IP_5_, or IP_6_ at 37°C for 15 min, and then at 55°C for 20 min. Beads were washed with lysis buffer (25 mM HEPES, pH 7.4, 1% NP-40, 0.1% BSA, 100 mM KCl, 150 mM NaCl, and protease and phosphatase inhibitor cocktails) and incubated overnight with 1 mg/ml HEK293T lysate (prepared in lysis buffer). Bound proteins were eluted in Laemmli buffer and were resolved by 10% SDS–PAGE. Following western transfer to a PVDF membrane, the proteins were detected by IB with anti-GST and anti-p150*^Glued^* antibodies. For coimmunoprecipitation of endogenous dynein IC and p150*^Glued^*, MEFs harvested from 10 cm dishes were lysed by shearing in lysis buffer without NP-40 or by solubilization for 30 min at 4°C in NP-40 containing lysis buffer. The lysate was centrifuged at 14 000 ***g*** for 20 min. Proteins were cross-linked for 30 min by the addition of 2.5 mM DSP, a thiol-cleavable cross-linker (22585, Thermo Scientific). The cross-linker was added either during cell lysis or subsequent to the removal of the cell debris, and cross-linking was quenched by adding 50 mM Tris–Cl, pH 7.4, for 15 min at 4°C. Two micrograms of anti-dynein IC or anti-p150*^Glued^* antibodies were added to the lysate and incubated overnight at 4°C. Protein A/G Dynabeads (88802, Thermo Scientific) were added to the complex and incubated at 4°C for 2 h. Proteins were eluted in Laemmli buffer containing 179 mM β-mercaptoethanol to reverse DSP cross-links and analyzed by IB with anti-dynein IC or anti-p150*^Glued^* antibodies.

### Crude membrane preparation

*Ip6k1*^+/+^ and *Ip6k1*^−/−^ MEFs grown in 150 mm dishes were scraped in 2 ml of fractionation buffer (25 mM HEPES, pH 7.4, 10 mM KCl, 1.5 mM MgCl_2_, 250 mM sucrose, 1 mM DTT, and protease inhibitor cocktail). Cells were lysed by passing through a 24-gauge needle (total homogenate, TH) and centrifuged at 10 000 ***g*** to pellet nuclei and mitochondria. The supernatant containing the cytosol and the membrane fraction (PNS) was subjected to high-speed centrifugation (Beckman) at 100 000 ***g*** for 1 h. The pellet containing the membrane fraction (membrane pellet, MP) was solubilized in 500 µl of fractionation buffer containing 1% Triton-X-100. The subcellular protein fractions were resolved on a 4–12% Nu-PAGE gel and analyzed by IB with anti-dynein IC, anti-p150*^Glued^*, anti-GM130, and anti-α-tubulin antibodies.

### Overexpression of dynein IC fragments

HeLa cells were grown on 12 mm coverslips in 24-well plates and 2 µg of pEYFP-Golgi (Clontech) was cotransfected with 2 µg of pCDNA3.1(+) or pCDNA-IC(1–111) or pCDNA-IC(1–111)S51A using polyethylenimine (Sigma-Aldrich). Forty-eight hours posttransfection, cells were fixed in 2% PFA. Coverslips were mounted in mounting medium containing DAPI, and confocal microscope images were acquired as described above.

### Statistics

Graphs and analyses were produced using GraphPad Prism 5. Continuous data were tested for normality and analyzed using one-way ANOVA, two-tailed unpaired Student's *t*-test, or two-tailed Mann–Whitney test, as appropriate. Fisher's exact test was used to assess categorical data presented as contingency tables. Statistical significance was defined as *P *< 0.05. The cell numbers used to obtain quantitative data (*n*) and the number of independent experiments performed are indicated in the figure legends.

## Results

### Endosomal sorting of Tfn in fibroblasts requires IP6K1 activity

**Figure 1.**
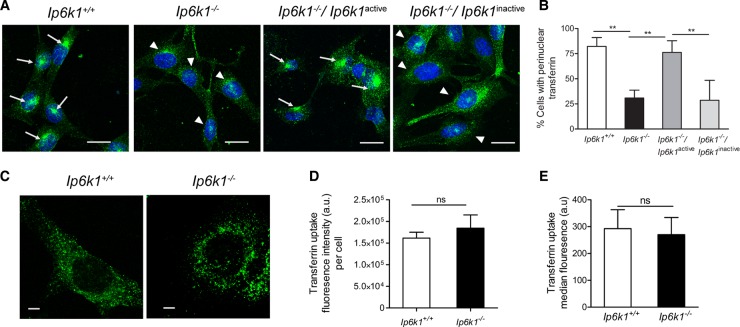
IP6K1 activity regulates Tfn trafficking. (**A**) MEFs of indicated genotypes were pulsed for 1 h with Alexa Fluor 488 Tfn (green), and the location of the endocytosed Tfn relative to the nucleus (blue) was examined. Arrows indicate cells showing Tfn accumulation in the perinuclear ERC and arrowheads indicate cells which do not show clear perinuclear Tfn accumulation. Scale bars, 20 µm. (**B**) The percentage of cells with Tfn accumulation in perinuclear ERC-like structures in (**A**) was calculated for each experiment. Data are mean ± SEM from three independent experiments (total number of cells analyzed were 174 and 232 cells, respectively, for *Ip6k1*^+/+^ and *Ip6k1*^−/−^ MEFs, and 154 and 285 cells, respectively, for *Ip6k1*^−/−^ MEFs expressing active or inactive forms of IP6K1). Data were analyzed using one-way ANOVA with Tukey's multiple comparison *post hoc* test; **P ≤ 0.01. (**C**) *Ip6k1*^+/+^ and *Ip6k1*^−/−^ MEFs were serum-starved for 1 h and pulsed with Alexa Fluor 488-labeled Tfn for 5 min. Scale bars, 5 µm. (**D**) Quantification of fluorescence intensity in (**C**), represented in arbitrary units (a.u.). Data (mean ± SEM, *n* = 55 and 51 cells, respectively, for *Ip6k1*^+/+^ and *Ip6k1*^−/−^ MEFs) are representative of three independent experiments and were analyzed using a two-tailed Mann–Whitney test; ns, not significant, *P *> 0.05. (**E**) Flow cytometry-based measurement of Alexa Fluor 488-labeled Tfn uptake in 5 min, represented in a.u.. Data (mean ± SEM of the median fluorescence from 10 000 cells) are compiled from three independent experiments and were analyzed using a two-tailed unpaired Student's *t*-test; ns, not significant, *P *> 0.05. Images in (**C**) were subjected to ‘levels’ adjustment in Adobe Photoshop to improve visualization.

To investigate the role of inositol pyrophosphates in dynein-dependent vesicle transport, we used MEFs derived from *Ip6k1*^−/−^ mice, in which IP_7_ levels are reduced to ∼30% compared with *Ip6k1*^+/+^ MEFs [20]. In interphase cells, dynein mediates endosomal sorting and Golgi organization [28]. To examine endosomal sorting, we tracked the localization of the iron-binding protein Tfn, which undergoes clathrin-dependent endocytosis and is transported from early endosomes to recycling endosomes in a dynein-dependent manner [28]. We allowed cells to take up fluorescently tagged Tfn for 1 h and scored for cells with accumulation of Tfn in the perinuclear ERC. The fraction of cells containing Tfn in ERC-like structures was significantly reduced in *Ip6k1*^−/−^ compared with *Ip6k1*^+/+^ MEFs (Figure 1A,B). When catalytically active IP6K1 was expressed in *Ip6k1*^−/−^ MEFs, it restored the subcellular distribution of Tfn, whereas expression of similar levels of the kinase-dead IP6K1 mutant enzyme (IP6K1^K226A/S334A^) [17] had no effect (Figure 1A,B). This suggests that the synthesis of inositol pyrophosphates by IP6K1 is required for efficient Tfn trafficking in cells. To rule out any defect in early endocytosis, we pulse-labeled cells with Tfn for 5 min and quantified the endocytosed Tfn by confocal microscopy and flow cytometry. Tfn uptake was found to be similar in both *Ip6k1*^+/+^ and *Ip6k1*^−/−^ MEFs, suggesting that endocytosis is normal in the absence of IP6K1 (Figure 1C–E).

**Figure 2.**
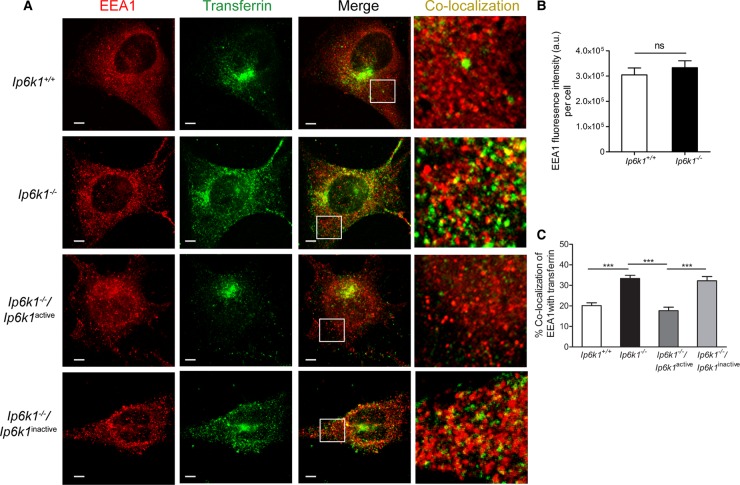
Tfn is held back in early endosomes in cells lacking IP6K1. (**A**) MEFs of indicated genotypes were pulsed with Alexa Fluor 488-labeled Tfn (green) for 1 h and immunostained with an antibody directed against the early endosome marker, EEA1 (red). Representative images show costaining of Tfn and EEA1. Scale bars, 5 µm. To visualize the overlap of Tfn with EEA1-positive structures distributed in the cytoplasm, the area within the white square in the merge panel is enlarged in the colocalization panel. (**B**) Quantification of EEA1 staining intensity in (**A**). Data (mean ± SEM, *n* = 58 and 77 cells, respectively, for *Ip6k1*^+/+^ and *Ip6k1*^−/−^ MEFs) are representative of two independent experiments and were analyzed using a two-tailed Mann–Whitney test; ns, not significant, *P *> 0.05. (**C**) Colocalization of EEA1-positive structures with Tfn calculated as the percentage of colocalized pixels with respect to the total number of EEA1-positive pixels per cell. Data (mean ± SEM; *n *= 58 and 77 cells, respectively, for *Ip6k1*^+/+^ and *Ip6k1*^−/−^ MEFs; *n* = 51 and 52 cells, respectively, for *Ip6k1*^−/−^ MEFs expressing active or inactive forms of IP6K1) are representative of two independent experiments and were analyzed using one-way ANOVA with Tukey's multiple comparison *post hoc* test; ***P ≤ 0.001. Images in (**A**) were subjected to ‘levels’ adjustment in Adobe Photoshop to improve visualization.

Decreased Tfn distribution in the ERC in *Ip6k1*^−/−^ MEFs could be due to a delay in Tfn trafficking from endosomes. To determine whether Tfn is held back in endosomes, we stained cells to detect the early endosome marker EEA1 1 h after Tfn uptake (Figure 2A). There is no change in EEA1 levels (Figure 2B), and it appears distributed throughout the cytoplasm in both cell types (Figure 2A). In *Ip6k1*^−/−^ MEFs, there is significant colocalization of endocytosed Tfn with EEA1-positive structures in the cytoplasm, whereas in *Ip6k1*^+/+^ MEFs, the early endosomes lack Tfn (Figure 2A, colocalization panel). To quantify these observations, we determined the percentage of Tfn that colocalizes with EEA1 throughout the cell. *Ip6k1*^−/−^ MEFs had significantly higher colocalization of Tfn in EEA1-containing vesicles when compared with *Ip6k1*^+/+^ MEFs (Figure 2A,C). This suggests that Tfn exit from early endosomes towards the ERC is slower in cells lacking IP6K1. Expression of active but not inactive IP6K1 rescued this defect, implying that inositol pyrophosphates are required for endosomal sorting of Tfn (Figure 2A,C).

### IP6K1 activity is required to maintain Golgi morphology

**Figure 3.**
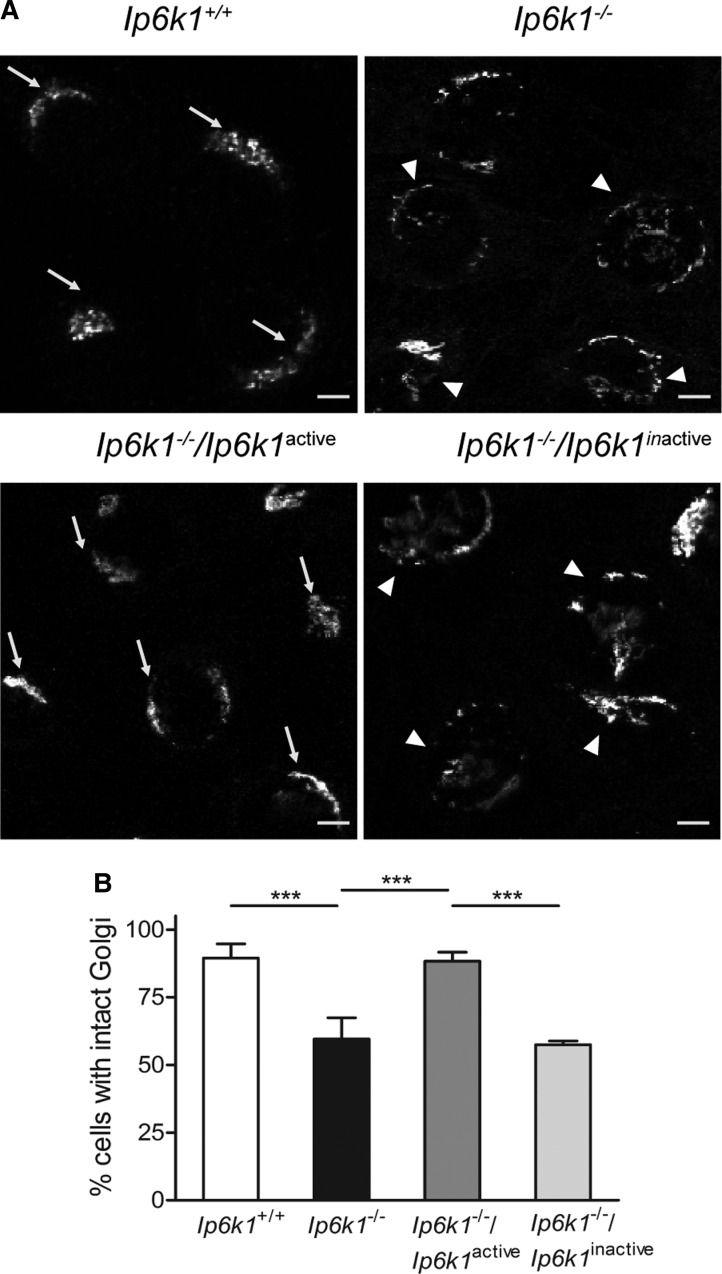
IP6K1 activity is required to maintain Golgi morphology. (**A**) MEFs of indicated genotypes were stained for GM130, a *cis*-Golgi marker. Arrows indicate cells with intact Golgi morphology and arrowheads indicate cells with fragmented Golgi. Scale bars, 20 µm. (**B**) Percentage of cells with intact Golgi morphology in (**A**) was calculated for each experiment. Data are mean ± SEM from three independent experiments (total number of cells analyzed were 100 and 103 cells, respectively, for *Ip6k1*^+/+^ and *Ip6k1*^−/−^ MEFs, and 88 and 92 cells, respectively, for *Ip6k1*^−/−^ MEFs expressing active or inactive forms of IP6K1). Data were analyzed using one-way ANOVA with Tukey's multiple comparison *post hoc* test; ***P ≤ 0.001.

Cytoplasmic dynein is required to position the Golgi apparatus in the pericentriolar region. In cells with defective dynein function, the Golgi appears fragmented [29]. When stained for the *cis*-Golgi marker GM130, most *Ip6k1*^+/+^ MEFs showed a normal perinuclear arc-like appearance of the Golgi, whereas a significant fraction of *Ip6k1*^−/−^ MEFs displayed a fragmented Golgi complex (Figure 3A,B). This is a hallmark phenotype of defective dynein function in mammalian cells. Expression of catalytically active but not inactive IP6K1 was able to correct the Golgi morphology in *Ip6k1*^−/−^ MEFs (Figure 3A,B), suggesting that the synthesis of inositol pyrophosphates is required for Golgi maintenance.

### Vesicle movement is slower in cells lacking IP6K1

**Figure 4.**
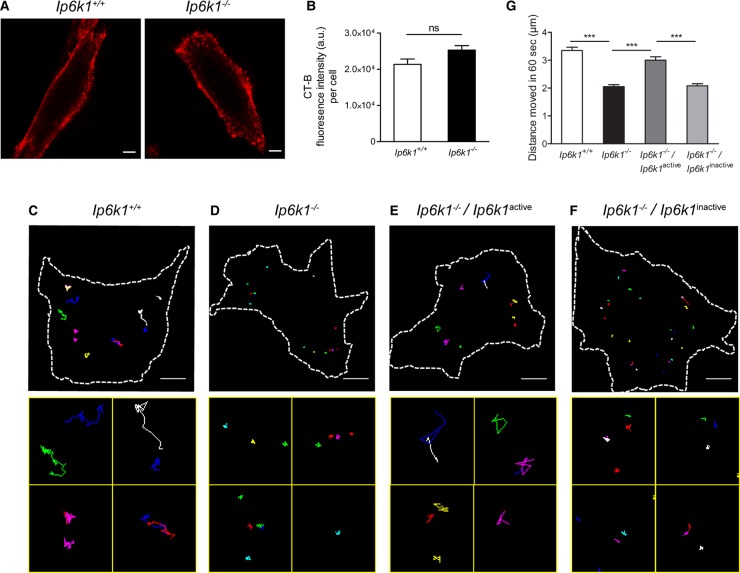
Slower vesicle motility in cells with reduced levels of inositol pyrophosphates. (**A**) Representative images of *Ip6k1*^+/+^ and *Ip6k1*^−/−^ MEFs bound to Alexa Fluor 594-conjugated CT-B. Scale bar, 5 μm. (**B**) Quantification of fluorescence intensity of CT-B per cell in (**A**) after 1 h binding on ice. Data (mean ± SEM, *n* = 25 cells for *Ip6k1*^+/+^ and *Ip6k1*^−/−^ MEFs) are representative of two independent experiments and were analyzed using a two-tailed Mann–Whitney test; ns, not significant, *P *> 0.05. (**C**–**F**) Representative tracks of Supplementary Videos S1–S4 of CT-B movement as analyzed using the manual tracking plugin in ImageJ (upper panel) and enlarged tracks from different fields within the same cell (lower panel). Scale bar, 10 μm. (**G**) Distance moved in 1 min by CT-B containing vesicles in MEFs of indicated genotypes (see Supplementary Videos S1–S4). Data (mean ± SEM; *n* = 111 and 102 vesicles, respectively, for *Ip6k1*^+/+^ and *Ip6k1*^−/−^ MEFs; *n* = 95 and 102 vesicles, respectively, for *Ip6k1*^−/−^ MEFs expressing active or inactive forms of IP6K1) are compiled from three independent experiments and were analyzed using one-way ANOVA with Tukey's multiple comparison *post hoc* test; ***P ≤ 0.001.

Defects in motor-dependent trafficking lead to impaired vesicle movement along microtubule tracks. To monitor vesicle motility, we used CT-B, which binds to the cell surface ganglioside GM1, and is endocytosed and trafficked to the trans-Golgi network [30]. We allowed fluorescently labeled CT-B to bind cells on ice for 1 h and noted no difference in CT-B binding to the cell surface in *Ip6k1*^+/+^ and *Ip6k1*^−/−^ MEFs (Figure 4A,B). Next, we allowed cells to endocytose CT-B for 30 min under regular culture conditions, and tracked the movement of fluorescent vesicles. We excluded endocytic vesicles that were close to the plasma membrane and perinuclear vesicles that are likely to have completed their movement towards the trans-Golgi network. The rate of transport of CT-B-containing endosomes is significantly lower in *Ip6k1*^−/−^ compared with *Ip6k1*^+/+^ MEFs (Figure 4C,D,G and Supplementary Videos S1 and S2). CT-B vesicle movement was restored upon expression of the catalytically active but not the kinase-dead form of IP6K1 (Figure 4E–G and Supplementary Videos S3 and S4), confirming that the intracellular levels of inositol pyrophosphates influence vesicle motility.

**Figure 5.**
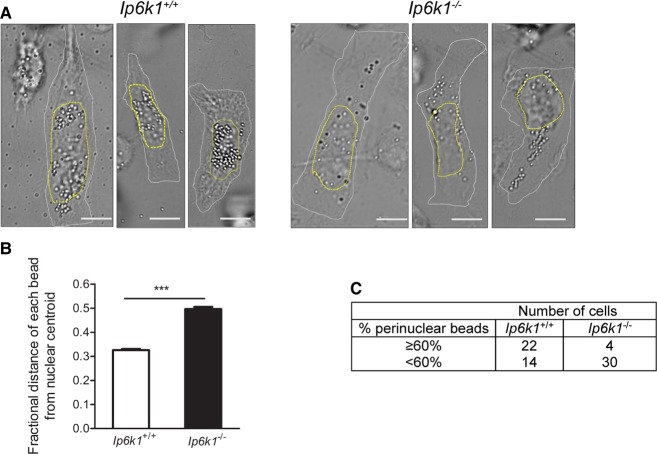
Phagosomal motility requires IP6K1. (**A**) Peritoneal macrophages isolated from *Ip6k1*^+/+^ and *Ip6k1*^−/−^ mice were pulsed with 750 nm latex beads for 1 h, washed and incubated in serum-containing medium for 1 h, and imaged by DIC microscopy to assess the localization of beads. The figure shows three representative cells of each genotype. The white dotted line indicates the cell outline and the yellow dotted line shows the nucleus outline. Scale bar, 10 μm. (**B**) The fractional distance of each phagocytosed bead from the nuclear centroid was calculated as described in the Materials and Methods section. Data (mean ± SEM; *n* = 910 beads in 36 cells for *Ip6k1*^+/+^ and 604 beads in 34 cells for *Ip6k1*^−/−^ macrophages) are compiled from two independent experiments and were analyzed using a two-tailed Mann–Whitney test; ***P ≤ 0.001. (**C**) Contingency table showing cell-based quantification of data in (**A**). Beads with a fractional distance of ≤0.4 were classified as ‘perinuclear’, and each cell was categorized as having either ≥60 or <60% perinuclear beads. Data were analyzed by a two-tailed Fisher's exact test, *P *≤ 0.001.

Dynein-dependent vesicle movement to the cell interior can be assessed by monitoring the intracellular distribution of phagosomes, which are transported along microtubules towards perinuclear lysosomes [31]. We isolated peritoneal macrophages from *Ip6k1*^+/+^ and *Ip6k1*^−/−^ mice, allowed them to phagocytose latex beads for 1 h, and observed their distribution 1 h postinternalization. Most of the beads were observed in the perinuclear region in *Ip6k1*^+/+^ macrophages, whereas in *Ip6k1*^−/−^ macrophages, a larger number of the beads were away from the nucleus (Figure 5A). Analysis of the fractional distance of each bead from the nuclear centroid revealed a greater nuclear proximity of phagosomes in *Ip6k1*^+/+^ macrophages compared with *Ip6k1*^−/−^ macrophages (Figure 5B,C). These data also reveal that the dependence of dynein on IP6K1 is conserved in cell types other than fibroblasts.

### Dynein-driven endosomal motility is reduced in slime mold lacking inositol pyrophosphates

To study the influence of cellular inositol pyrophosphates on motility at the level of individual vesicles, we turned to the slime mold *D. discoideum*. Since the cellular functions of dynein in *D. discoideum* are highly similar to those seen in mammalian cells [32], this slime mold has been extensively used to examine dynein-dependent motor activity *in vitro* [23]. *D. discoideum* has 200–500-fold higher levels of inositol pyrophosphates compared with mammalian cells [4,18]. Deletion of the IP_6_ kinase *i6kA* in the amoeba results in the absence of any detectable cellular inositol pyrophosphates [18]. We assessed the *in vitro* motility of endosomes derived from wild-type and *i6kA*^−^ *D. discoideum* on polarity-labeled microtubules [23]. As these microtubules are polymerized *in vitro* using tubulin purified from goat brain, this assay rules out any influence of inositol pyrophosphates on microtubule assembly or its posttranslational modifications. The fraction of motile endosomes (that were a mix of plus- and minus-end moving) was not significantly different in wild-type and *i6kA*^−^ amoebae (Table 1). However, the percentage of dynein-driven minus-end-directed motile endosomes was substantially lower in *i6kA*^−^ *D. discoideum.* The apparent increase in kinesin-dependent plus-end-directed motile endosomes is most likely a consequence of reduced dynein-driven motility, as both classes of motor proteins are recruited onto endosomes and are reported to engage in a mechanical tug-of-war [24]. We observed no change in the velocity of minus-end- or plus-end-directed endosomes, suggesting that single-molecule properties and ATPase activity of both motor proteins are not compromised in *i6kA*^−^ amoebae. These observations confirm that inositol pyrophosphates regulate dynein-dependent vesicle movement even in lower eukaryotes such as slime mold.

**Table 1:** Motility of endosomes derived from *D. discoideum* on polarity-labeled microtubules *In vitro* motility of refractile endosomes in PNS from wild-type and *i6kA*^−^ *D. discoideum* was assayed on polarity-labeled microtubules. Data were compiled from two independent experiments. For statistical analysis, the endosome velocities (mean ± SEM) were analyzed by a two-tailed Student's *t*-test. Contingency tables (2 × 2) of the number of motile vs. nonmotile endosomes and of the number of plus-end- vs. minus-end-directed endosomes were subjected to a two-tailed Fisher's exact test. The number of endosomes used to measure velocity is indicated in brackets. The observed motion of each endosome was parsed into multiple velocity segments by a Bayesian algorithm.

Parameters analyzed	Wild type	*i6kA*^−^	Significance (*P*)
Number of endosomes studied	72	89	
Motile fraction (%)	76	64	>0.05
Minus-end-directed movement (%)	76	39	<0.001
Plus-end-directed movement (%)	24	61	
Minus-end-directed velocity (µm/s)	1.99 ± 0.10 (9)	1.94 ± 0.63 (15)	>0.05
Plus-end-directed velocity (µm/s)	2.08 ± 0.18 (16)	1.93 ± 0.13 (10)	>0.05

### Dynein IC is pyrophosphorylated by 5-IP_7_

**Figure 6.**
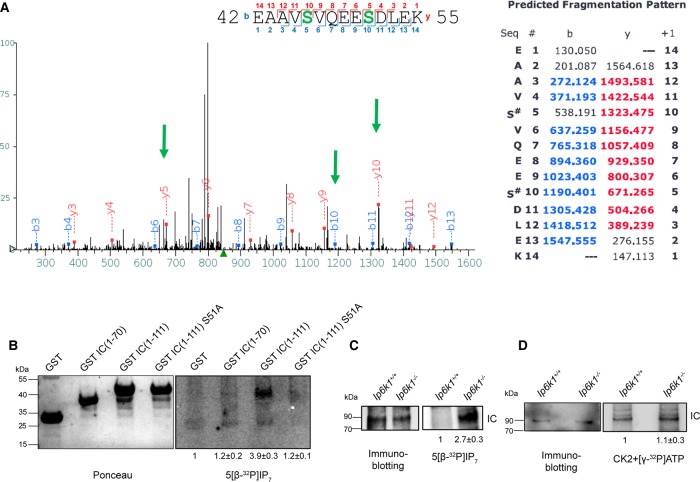
Inositol pyrophosphates pyrophosphorylate dynein IC. (**A**) Bacterially expressed and purified IC(1–111) was phosphorylated *in vitro* by CK2, and phosphosite identification was contracted out to the Taplin Mass Spectrometry Facility, Harvard Medical School. The MS/MS spectrum is shown for the doubly phosphorylated peptide corresponding to residues 42–55 of mouse IC-2C (EAAVpSVQEEpSDLEK). The sequence shows the peptide fragmentation pattern, and the table shows masses of all b and y ions, highlighting the ions obtained in the spectrum. Arrows indicate fragment ions containing phosphorylated Ser residues. The mass of fragment y5 indicates phosphorylation of Ser51, and the masses of y10 and b10 correspond to phosphorylation of Ser46 and Ser51. (**B**) Bacterially expressed and purified GST or GST-tagged IC(1–70), IC(1–111), and IC(1–111)S51A were prephosphorylated with CK2 and unlabeled ATP and incubated with 5[β-^32^P]IP_7_. Proteins were resolved using NuPAGE and transferred to a PVDF membrane. Pyrophosphorylation was detected by phosphorimager scanning (right) and the proteins were detected by Ponceau S staining (left). The phosphorimager scan was subjected to ‘levels’ adjustment in Adobe Photoshop to improve visualization. The image intensity of the pyrophosphorylated protein was normalized to the corresponding total protein. The pyrophosphorylation intensity of each IC fragment was compared with GST. Data are mean ± range from two independent experiments. (**C**) Back-pyrophosphorylation of endogenous dynein IC by IP_7_. Dynein IC immunoprecipitated from *Ip6k1*^+/+^ and *Ip6k1*^−/−^ MEFs was incubated with 5[β-^32^P]IP_7_, resolved by NuPAGE, and transferred to a PVDF membrane. Pyrophosphorylation was detected by phosphorimager scanning (right), and proteins were detected by western blotting (left). The image intensity of the pyrophosphorylated protein was normalized to the corresponding immunoprecipitated protein. The fold change in the extent of pyrophosphorylation of IC in *Ip6k1*^−/−^ compared with *Ip6k1*^+/+^ MEFs is indicated. Data are mean ± SEM from three independent experiments. (**D**) Back-phosphorylation of endogenous dynein IC by CK2. Dynein IC immunoprecipitated from *Ip6k1*^+/+^ and *Ip6k1*^−/−^ MEFs was incubated with CK2 and [γ-^32^P]ATP. Proteins were resolved and detected as in (**C**). The fold change in phosphorylation was calculated as in (**C**). Data are mean ± range from two independent experiments.

Our data so far show that lowering cellular levels of inositol pyrophosphates leads to reduced dynein-dependent vesicle transport. The dynein complex contains two large catalytic heavy chains that move the motor along microtubules and several small noncatalytic subunits that function in vesicle attachment and dynein structure maintenance [13]. One mechanism by which inositol pyrophosphates may directly influence dynein function is by serine pyrophosphorylation on dynein subunits. A consensus pyrophosphorylation site comprises one or more Ser residues flanked by Asp/Glu residues [9–11,33]. This is also a preferred site for phosphorylation by the protein kinase CK2, which is known to prephosphorylate the target Ser to prime it for pyrophosphorylation [9]. CK2 has been shown to phosphorylate the N-terminus of the dynein IC-2C [34,35], a noncatalytic dynein subunit, which possesses multiple phosphorylation sites, including a pyrophosphorylation consensus sequence (Supplementary Figure S1). The N-terminus of IC-2C, the IC isoform expressed in all tissues [19], is intrinsically disordered [36,37] and can be divided into a charge cluster and a Ser-Pro-rich region (Supplementary Figure S1). To identify the sites of CK2 phosphorylation on the IC-2C N-terminus, we expressed the N-terminal 111 amino acid residues of mouse IC-2C as a fusion to GST and phosphorylated it with CK2 *in vitro*. Mass spectrometry revealed two sites of CK2 phosphorylation in the charge cluster (Ser46 and Ser51) and one site in the Ser-Pro cluster (Ser98; Figure 6A and Supplementary Figure S2). All three sites have been shown to be endogenously phosphorylated (Supplementary Figure S1). From amongst these three CK2 sites, Ser51 lies amidst Glu and Asp residues and constitutes a preferred site for pyrophosphorylation [9].

To test for IP_7_-mediated pyrophosphorylation, we incubated CK2-prephosphorylated IC fragments, IC(1–70) encompassing the charge cluster, and IC(1–111) which includes the charge and Ser-Pro clusters, with radiolabeled IP_7_. We observed that IC(1–111) undergoes pyrophosphorylation, whereas IC(1–70), which harbors the consensus IP_7_ target site, is not pyrophosphorylated (Figure 6B). Interestingly, mutating the consensus IP_7_ site by replacing Ser51 with Ala abolished pyrophosphorylation in IC(1–111) (Figure 6B), implying that Ser51 is indeed the target of IP_7_. The absence of pyrophosphorylation in the IC(1–70) fragment suggests that the Ser-Pro cluster (residues 71–111) is required to facilitate pyrophosphorylation on Ser51. Interestingly, the site of pyrophosphorylation we have identified in mouse IC-2C is well conserved in human and rat (Supplementary Figure S1), suggesting that the effect of IP_7_ on dynein is likely to be conserved in these species. In *D. discoideum*, the IP_7_ target Ser is conserved, but the neighboring Asp and Glu residues are replaced with Thr. These Thr residues may undergo phosphorylation to mimic Asp/Glu and create a consensus site for pyrophosphorylation.

To assess whether IC undergoes pyrophosphorylation *in vivo*, we carried out a ‘back-pyrophosphorylation’ assay [8,10]. This is currently the accepted method to assess IP_7_-mediated pyrophosphorylation *in vivo*, as mass spectrometry, the preferred method for detection of phosphorylation, cannot distinguish between pyro- versus bisphosphorylated peptides [38]. As an IP_7_ target protein isolated from *Ip6k1*^+/+^ MEFs is already pyrophosphorylated *in vivo*, it has been shown to display diminished incorporation of radiolabeled phosphate from IP_7_ *in vitro* [10]. On the other hand, the same target protein isolated from *Ip6k1*^−/−^ MEFs displays augmented pyrophosphorylation *in vitro* due to reduced pyrophosphorylation *in vivo*. Endogenous IC immunoprecipitated from *Ip6k1*^+/+^ and *Ip6k1*^−/−^ MEFs was incubated with radiolabeled IP_7_ without pre-phosphorylation by CK2 and examined for the extent of radiolabeled phosphate transfer. We observed no 5[β^32^P]-IP_7_-mediated pyrophosphorylation of native IC from *Ip6k1*^+/+^ MEFs (Figure 6C), suggesting that this protein is heavily pyrophosphorylated *in vivo*. Conversely, IC from *Ip6k1*^−/−^ MEFs was robustly pyrophosphorylated *in vitro*, implying that loss of IP6K1 leads to diminished pyrophosphorylation of IC inside cells (Figure 6C). It has been previously suggested that lower ‘back-pyrophosphorylation’ of a native protein by radiolabeled IP_7_ in wild-type cells could also be due to lower endogenous CK2-mediated pre-phosphorylation [39]. To address this possibility, we performed a CK2 ‘back-phosphorylation’ assay, in which immunoprecipitated IC from *Ip6k1*^+/+^ and *Ip6k1*^−/−^ MEFs was incubated with radiolabeled ATP in the presence of CK2. We observed no significant difference in the incorporation of phosphate from ATP (Figure 6D). While any variation in phosphorylation of individual CK2 target residues on native IC-2C is unclear, this result suggests that the overall extent of endogenous CK2 phosphorylation is similar in *Ip6k1*^+/+^ and *Ip6k1*^−/−^ MEFs.

### Pyrophosphorylation of IC positively regulates its interaction with p150*^Glued^*

**Figure 7.**
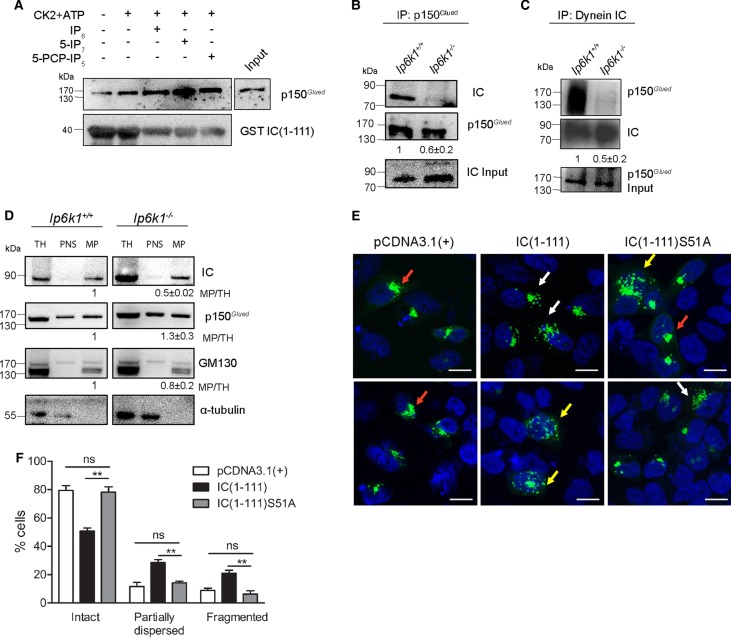
Pyrophosphorylation of IC regulates its interaction with p150^*Glued*^. (**A**) Blots representative of two independent experiments showing the effect of IP_6_, 5-IP_7_, or 5-PCP-IP_5_ on the ability of prephosphorylated GST IC(1–111) to pull down endogenous p150*^Glued^* from HEK293T cell lysates. (**B** and **C**) Coimmunoprecipitation of dynein IC and p150*^Glued^* from *Ip6k1*^+/+^ and *Ip6k1*^−/−^ MEFs. Protein extracts were cross-linked with a thiol-cleavable cross-linker, followed by immunoprecipitation of p150*^Glued^* and IC. Representative immunoblots of coimmunoprecipitation of IC with p150*^Glued^* (**B**) and p150*^Glued^* with IC (**C**). The levels of coimmunoprecipitated IC or p150*^Glued^* were normalized to the level of the immunoprecipitated partner. The fold change in the extent of coimmunoprecipitation in *Ip6k1*^−/−^ compared with *Ip6k1*^+/+^ MEFs is indicated as mean ± SEM from three (**B**) and four (**C**) independent experiments. A higher extent of cross-linking led to more smearing of bands in (**C**) compared with (**B**) due to differences in the methods used for lysis. (**D**) Representative blots of subcellular fractions from *Ip6k1*^+/+^ and *Ip6k1*^−/−^ MEFs prepared by differential centrifugation were resolved on a 4–12% NuPAGE gel and immunoblotted to detect dynein IC and p150*^Glued^* in TH, PNS, and MP. An increased amount of protein was loaded in the *Ip6k1*^−/−^ fractions to enable visualization of the dynein IC. GM130 was used as a membrane marker and α-tubulin was used as a loading control. The level of each protein in the MP fraction was normalized to its levels in TH. The fold change in protein levels in *Ip6k1*^−/−^ compared with *Ip6k1*^+/+^ MEFs is indicated below each blot. Data are mean ± SEM from three independent experiments. (**E**) HeLa cells were cotransfected with EYFP-Golgi (green) and vector control or IC(1–111) or IC(1–111)S51A, and nuclei were stained with DAPI (blue). Two representative fields from the same experiment are shown. Red arrows indicate intact Golgi, white arrows indicate partially dispersed Golgi, and yellow arrows indicate fragmented Golgi morphology. (**F**) Quantification of (**E**). EYFP-positive cells were categorized as shown in (**E**) according to their Golgi morphology, and the percentage of cells in each category was calculated for each experiment. Data are mean ± SEM of four independent experiments (total EYFP-positive cells analyzed were 154 for control cells; 241 and 194 for cells overexpressing IC(1–111) and IC(1–111)S51A, respectively). Data were analyzed using one-way ANOVA with Tukey's multiple comparison *post hoc* test; **P ≤ 0.01.

Dynein IC facilitates dynein recruitment to vesicles via its interaction with the p150*^Glued^* subunit of the dynactin complex [40,41]. The first 106 residues in the N-terminal region of IC-2C contain binding sites for the CC1 domain of p150*^Glued^* [41]. To test the effect of IC pyrophosphorylation on its interaction with p150*^Glued^*, phosphorylated and pyrophosphorylated mouse IC(1–111) were assessed for their ability to pull down native p150*^Glued^* from human HEK293T cell lysates. Human and mouse dynein IC-2C show 95% sequence identity in the N-terminal region, and the CC1 region of p150*^Glued^* is 99% identical, suggesting that the regulation of dynein–dynactin interaction is likely to be conserved in these species. IC(1–111) that was CK2-phosphorylated and then pyrophosphorylated by 5-IP_7_ shows enhanced interaction with p150*^Glued^* compared with the CK2-phosphorylated fragment alone (Figure 7A). IP_6_ or a nonhydrolyzable analog of 5-IP_7_ (5-PCP-IP_5_) [42], which is likely to bind but cannot pyrophosphorylate the target protein, induces only a marginal enhancement in p150*^Glued^* binding to IC(1–111) (Figure 7A).

To examine whether the loss of pyrophosphorylation on IC in *Ip6k1*^−/−^ MEFs alters its interaction with p150*^Glued^*, we conducted coimmunoprecipitation assays after cross-linking proteins with a reversible cross-linker. We noted a significant decrease in the extent of IC and p150*^Glued^* interaction in extracts from *Ip6k1*^−/−^ MEFs compared with *Ip6k1*^+/+^ MEFs (Figure 7B,C). To monitor if the decrease in IC–p150*^Glued^* interaction leads to reduced dynein recruitment on vesicle membranes, extracts from *Ip6k1*^+/+^ and *Ip6k1*^−/−^ MEFs were subjected to differential centrifugation to isolate the membrane fraction. The amounts of IC, p150*^Glued^*, and the Golgi matrix protein GM130 in the membrane fraction were normalized to their levels in the TH. The ratio of IC in the MP, compared with total cell homogenate, was significantly lower in *Ip6k1*^−/−^ MEFs compared with *Ip6k1*^+/+^ MEFs (Figure 7D). In contrast, the ratios of p150*^Glued^* and GM130 were unchanged in the same extracts.

Earlier studies have shown that overexpression of the IC N-terminal region in HeLa cells produces a dominant-negative effect by binding to p150*^Glued^* and preventing its interaction with the endogenous dynein complex, leading to Golgi disruption [41]. Therefore, we hypothesized that overexpression of the IC(1–111) wild-type fragment should disrupt Golgi morphology as it is capable of pyrophosphorylation and dynactin binding, whereas the S51A mutant, which cannot be pyrophosphorylated, should not exhibit Golgi fragmentation due to its reduced binding to p150*^Glued^*. IC(1–111) fragments were cotransfected with the enhanced YFP-tagged trans-Golgi marker β1,4-galactosyltransferase (EYFP-Golgi) [41], and the percentage of Golgi disruption was scored. The cells were categorized into intact Golgi, partially dispersed Golgi if more than three fragments were seen, and completely dispersed Golgi if the EYFP signal was scattered across the cytoplasm (Figure 7E). Approximately 50% of cells transfected with IC(1–111) showed intact Golgi, whereas 30 and 20% cells exhibited partially dispersed and completely fragmented Golgi, respectively (Figure 7F). In contrast, overexpression of IC(1–111)S51A was unable to disrupt the Golgi apparatus, as the Golgi distribution pattern in these cells was comparable to the vector control (Figure 7E,F). These results suggest that pyrophosphorylation of the dynein IC is required for effective binding of IC to p150*^Glued^* inside cells.

## Discussion

Our study has identified inositol pyrophosphates as novel regulators of dynein function in protozoan and metazoan cells. Inositol pyrophosphate-mediated serine pyrophosphorylation of the dynein IC promotes its interaction with the p150*^Glued^* subunit of dynactin, suggesting that pyrophosphorylation of IC may regulate attachment of the dynein motor to vesicle membranes. Corroborating this, we observed a decrease in IC–p150*^Glued^* association and decreased dynein binding to membranes in cells lacking IP6K1, leading to multiple defects in dynein-dependent vesicle transport in these cells. In fact, the phenotypic defects in Golgi maintenance and Tfn sorting observed in *Ip6k1*^−/−^ MEFs are similar to the phenotypes observed in dynein IC knockdown cells [28].

The dynein and dynactin protein assemblies interact at multiple sites, including a recently elucidated association of the dynein heavy chain with the Arp filament of dynactin, which is stabilized by the cargo adaptor protein Bicaudal-D2 [43,44]. However, the best characterized direct association between the dynein and dynactin complexes is the interaction between the coiled-coil region of p150*^Glued^* and the N-terminus of dynein IC [40,41]. In the absence of dynactin, the N-terminus of rat IC-2C, which is identical with mouse IC-2C (Supplementary Figure S1), is monomeric and disordered [37]. Residues 10–44 lying within this region constitute a core binding site for the coiled-coil CC1 domain of p150*^Glued^*. Two IC monomers bind a CC1 dimer to form a tetrameric complex held together predominantly by electrostatic interactions [37]. Given that disordered sequences are prone to posttranslational modifications, phosphorylation on residues in IC-2C surrounding the core binding site could influence its interaction with p150*^Glued^* [36,45]. Phosphorylation of Ser84 and Thr89 inhibits IC–p150*^Glued^* interaction, whereas phosphorylated Ser81 and Ser83 have no effect, and the role of phosphorylation at other sites (Supplementary Figure S1) is unknown [35,46,47]. Earlier work documenting CK2 phosphorylation of the IC-2C N-terminus did not identify the Ser residues phosphorylated by CK2 and was therefore unable to discern its influence on IC–p150*^Glued^* interaction [34,35]. Here, we identified three Ser residues in the IC-2C N-terminus that are phosphorylated by CK2 (Figure 6A and Supplementary Figure S2), but observe no effect of CK2 phosphorylation on its interaction with full-length p150*^Glued^* (Figure 7A). On the other hand, subsequent IP_7_-mediated pyrophosphorylation of Ser51, which lies in close proximity to the core p150*^Glued^*-binding region, significantly enhanced the binding of IC(1–111) to p150*^Glued^* (Figure 7A). Thus, pyrophosphorylation of dynein IC serves to stabilize IC–p150*^Glued^* association (Figure 7A–C), switching dynein from an unbound to a membrane-bound state (Figure 7D). Increasing membrane recruitment of multiple dynein motors would counteract the effect of kinesin, which is known to contest with dynein to regulate the directionality of organelle movement along microtubules [24]. Therefore, IC pyrophosphorylation may act as a regulatory signal to enhance minus-end-directed vesicle motility.

Pyrophosphorylation of the cargo adaptor protein subunit AP3B1 [10] and the yeast glycolytic transcription factor GCR1 [11] inhibits interaction with their respective binding partners, whereas we report augmented p150*^Glued^* binding as a consequence of dynein IC pyrophosphorylation, suggesting that this posttranslational modification can influence protein–protein interaction in diverse ways. Our study also sheds light on the mechanism of protein pyrophosphorylation, revealing that a disordered protein sequence distinct from the target Ser residue is required for pyrophosphorylation. Similarly, pyrophosphorylation target sites identified in three different yeast RNA Pol I subunits also fall within highly mobile regions [33], implying that flexible secondary structures promote phosphotransfer from IP_7_ to phosphoserine. As with other posttranslational modifications, pyrophosphorylation has recently been shown to be reversible [48], suggesting that dynein recruitment to vesicles via IC–p150*^Glued^* interaction may be regulatable by yet to be identified signaling pathways.

## Abbreviations

5-IP_7_, 5-diphosphoinositol pentakisphosphate; Alexa488 Tfn, Alexa Fluor 488-conjugated transferrin; AP3, adaptor protein 3; a.u., arbitrary units; CT-B, cholera toxin B subunit; DIC, differential interference contrast; DMEM, Dulbecco's modified Eagle's medium; DSP, dithiobis(succinimidyl propionate); ERC, endocytic recycling compartment; EYFP, enhanced yellow fluorescent protein; GST, glutathione S-transferase; I6KA, *Ip6k* in *Dictyostelium discoideum*; IB, immunoblotting; IC, dynein intermediate chain; IC-2C, dynein intermediate chain 2C; IF, immunofluorescence; IP_6_, inositol hexakisphosphate; IP6K1, inositol hexakisphosphate kinase 1; IP_7_, diphosphoinositol pentakisphosphate; MEFs, mouse embryonic fibroblasts; MP, membrane pellet; PFA, paraformaldehyde; PNS, postnuclear supernatant; Tfn, transferrin; TH, total homogenate.

## Author Contribution

M.C. performed most of the experimental work. A.R. and R.M. performed assays with *D. discoideum* endosomes. A.B.M. performed the membrane preparation experiment. M.W. and D.F. generated unlabeled 5-IP_7_ and 5-PCP-IP_5_. M.C. and R.B. designed the research and wrote the paper. All the authors read and agreed on the final version of the manuscript.

## Funding

This work was supported by the Wellcome Trust/Department of Biotechnology India Alliance [WT/DBT IA, 500020/Z/09/Z] and Centre for DNA Fingerprinting and Diagnostics core funds. M.C. and A.B.M are recipients of Junior and Senior Research Fellowships from the Council of Scientific and Industrial Research and the University Grants Commission, Government of India, respectively. D.F. acknowledges Princeton University start-up funds. R.M. acknowledges funding through an International Senior Research Fellowship from the Wellcome Trust UK [WT079214MA] and a WT/DBT IA Senior Fellowship [IA/S/11/2500255].

## References

[1] Stephens L., Radenberg T., Thiel U., Vogel G., Khoo K.H., Dell A. et al. (1993) The detection, purification, structural characterization, and metabolism of diphosphoinositol pentakisphosphate(s) and bisdiphosphoinositol tetrakisphosphate(s). J. Biol. Chem. 268, 4009–4015 PMID: 8440693

[2] Menniti F.S., Miller R.N., Putney J.W. Jr and Shears S.B. (1993) Turnover of inositol polyphosphate pyrophosphates in pancreatoma cells. J. Biol. Chem. 268, 3850–3856 PMID: 8382679

[3] Thota S.G. and Bhandari R. (2015) The emerging roles of inositol pyrophosphates in eukaryotic cell physiology. J. Biosci. 40, 593–605 doi:10.1007/s12038-015-9549-x 26333405

[4] Wundenberg T. and Mayr G.W. (2012) Synthesis and biological actions of diphosphoinositol phosphates (inositol pyrophosphates), regulators of cell homeostasis. Biol. Chem. 393, 979–998 doi:10.1515/hsz-2012-0133 22944697

[5] Saiardi A., Erdjument-Bromage H., Snowman A.M., Tempst P. and Snyder S.H. (1999) Synthesis of diphosphoinositol pentakisphosphate by a newly identified family of higher inositol polyphosphate kinases. Curr. Biol. 9, 1323–1326 doi:10.1016/S0960-9822(00)80055-X 10574768

[6] Saiardi A., Nagata E., Luo H.R., Snowman A.M. and Snyder S.H. (2001) Identification and characterization of a novel inositol hexakisphosphate kinase. J. Biol. Chem. 276, 39179–39185 doi:10.1074/jbc.M106842200 11502751

[7] Thomas M.P. and Potter B.V.L. (2014) The enzymes of human diphosphoinositol polyphosphate metabolism. FEBS J. 281, 14–33 doi:10.1111/febs.12575 24152294 PMC4063336

[8] Saiardi A., Bhandari R., Resnick A.C., Snowman A.M. and Snyder S.H. (2004) Phosphorylation of proteins by inositol pyrophosphates. Science 306, 2101–2105 doi:10.1126/science.1103344 15604408

[9] Bhandari R., Saiardi A., Ahmadibeni Y., Snowman A.M., Resnick A.C., Kristiansen T.Z. et al. (2007) Protein pyrophosphorylation by inositol pyrophosphates is a posttranslational event. Proc. Natl Acad. Sci. USA 104, 15305–15310 doi:10.1073/pnas.0707338104 17873058 PMC2000531

[10] Azevedo C., Burton A., Ruiz-Mateos E., Marsh M. and Saiardi A. (2009) Inositol pyrophosphate mediated pyrophosphorylation of AP3B1 regulates HIV-1 Gag release. Proc. Natl Acad. Sci. USA 106, 21161–21166 doi:10.1073/pnas.0909176106 19934039 PMC2795533

[11] Szijgyarto Z., Garedew A., Azevedo C. and Saiardi A. (2011) Influence of inositol pyrophosphates on cellular energy dynamics. Science 334, 802–805 doi:10.1126/science.1211908 22076377

[12] Hammer J.A. III and Sellers J.R. (2011) Walking to work: roles for class V myosins as cargo transporters. Nat. Rev. Mol. Cell. Biol. 13, 13–26 PMID: 22146746 10.1038/nrm3248

[13] Vallee R.B., McKenney R.J. and Ori-McKenney K.M. (2012) Multiple modes of cytoplasmic dynein regulation. Nat. Cell Biol. 14, 224–230 doi:10.1038/ncb2420 22373868

[14] Saiardi A., Caffrey J.J., Snyder S.H. and Shears S.B. (2000) The inositol hexakisphosphate kinase family: catalytic flexibility and function in yeast vacuole biogenesis. J. Biol. Chem. 275, 24686–24692 doi:10.1074/jbc.M002750200 10827188

[15] Saiardi A., Sciambi C., McCaffery J.M., Wendland B. and Snyder S.H. (2002) Inositol pyrophosphates regulate endocytic trafficking. Proc. Natl Acad. Sci. USA 99, 14206–14211 doi:10.1073/pnas.212527899 12391334 PMC137862

[16] Illies C., Gromada J., Fiume R., Leibiger B., Yu J., Juhl K. et al. (2007) Requirement of inositol pyrophosphates for full exocytotic capacity in pancreatic β cells. Science 318, 1299–1302 doi:10.1126/science.1146824 18033884

[17] Jadav R.S., Chanduri M.V.L., Sengupta S. and Bhandari R. (2013) Inositol pyrophosphate synthesis by inositol hexakisphosphate kinase 1 is required for homologous recombination repair. J. Biol. Chem. 288, 3312–3321 doi:10.1074/jbc.M112.396556 23255604 PMC3561551

[18] Pisani F., Livermore T., Rose G., Chubb J.R., Gaspari M. and Saiardi A. (2014) Analysis of *Dictyostelium discoideum* inositol pyrophosphate metabolism by gel electrophoresis. PLoS ONE 9, e85533 doi:10.1371/journal.pone.0085533 24416420 PMC3887064

[19] Kuta A., Deng W., Morsi El-Kadi A., Banks G.T., Hafezparast M., Pfister K.K. et al. (2010) Mouse cytoplasmic dynein intermediate chains: identification of new isoforms, alternative splicing and tissue distribution of transcripts. PLoS ONE 5, e11682 doi:10.1371/journal.pone.0011682 20657784 PMC2908135

[20] Bhandari R., Juluri K.R., Resnick A.C. and Snyder S.H. (2008) Gene deletion of inositol hexakisphosphate kinase 1 reveals inositol pyrophosphate regulation of insulin secretion, growth, and spermiogenesis. Proc. Natl Acad. Sci. USA 105, 2349–2353 doi:10.1073/pnas.0712227105 18268345 PMC2268139

[21] Rapaport D., Auerbach W., Naslavsky N., Pasmanik-Chor M., Galperin E., Fein A. et al. (2006) Recycling to the plasma membrane is delayed in EHD1 knockout mice. Traffic 7, 52–60 doi:10.1111/j.1600-0854.2005.00359.x 16445686

[22] Caviston J.P., Ross J.L., Antony S.M., Tokito M. and Holzbaur E.L.F. (2007) Huntingtin facilitates dynein/dynactin-mediated vesicle transport. Proc. Natl Acad. Sci. USA 104, 10045–10050 doi:10.1073/pnas.0610628104 17548833 PMC1891230

[23] Soppina V., Rai A. and Mallik R. (2009) Simple non-fluorescent polarity labeling of microtubules for molecular motor assays. BioTechniques 46, 543–549 doi:10.2144/000113124 19594454

[24] Soppina V., Rai A.K., Ramaiya A.J., Barak P. and Mallik R. (2009) Tug-of-war between dissimilar teams of microtubule motors regulates transport and fission of endosomes. Proc. Natl Acad. Sci. USA 106, 19381–19386 doi:10.1073/pnas.0906524106 19864630 PMC2770008

[25] Romero-Calvo I., Ocón B., Martínez-Moya P., Suárez M.D., Zarzuelo A., Martínez-Augustin O. et al. (2010) Reversible Ponceau staining as a loading control alternative to actin in Western blots. Anal. Biochem. 401, 318–320 doi:10.1016/j.ab.2010.02.036 20206115

[26] Shevchenko A., Wilm M., Vorm O. and Mann M. (1996) Mass spectrometric sequencing of proteins from silver-stained polyacrylamide gels. Anal. Chem. 68, 850–858 doi:10.1021/ac950914h 8779443

[27] Beausoleil S.A., Villén J., Gerber S.A., Rush J. and Gygi S.P. (2006) A probability-based approach for high-throughput protein phosphorylation analysis and site localization. Nat. Biotechnol. 24, 1285–1292 doi:10.1038/nbt1240 16964243

[28] Palmer K.J., Hughes H. and Stephens D.J. (2009) Specificity of cytoplasmic dynein subunits in discrete membrane-trafficking steps. Mol. Biol. Cell 20, 2885–2899 doi:10.1091/mbc.E08-12-1160 19386764 PMC2695796

[29] Corthesy-Theulaz I., Pauloin A. and Pfeffer S.R. (1992) Cytoplasmic dynein participates in the centrosomal localization of the Golgi complex. J. Cell Biol. 118, 1333–1345 doi:10.1083/jcb.118.6.1333 1387874 PMC2289611

[30] Lencer W.I. and Tsai B. (2003) The intracellular voyage of cholera toxin: going retro. Trends Biochem. Sci. 28, 639–645 doi:10.1016/j.tibs.2003.10.002 14659695

[31] Rai A.K., Rai A., Ramaiya A.J., Jha R. and Mallik R. (2013) Molecular adaptations allow dynein to generate large collective forces inside cells. Cell 152, 172–182 doi:10.1016/j.cell.2012.11.044 23332753

[32] Ma S., Fey P. and Chisholm R.L. (2001) Molecular motors and membrane traffic in *Dictyostelium*. Biochim. Biophys. Acta, Gen. Subj. 1525, 234–244 doi:10.1016/S0304-4165(01)00109-X 11257437

[33] Thota S.G., Unnikannan C.P., Thampatty S.R., Manorama R. and Bhandari R. (2015) Inositol pyrophosphates regulate RNA polymerase I-mediated rRNA transcription in *Saccharomyces cerevisiae*. Biochem. J. 466, 105–114 doi:10.1042/BJ20140798 25423617 PMC4325516

[34] Karki S., Tokito M.K. and Holzbaur E.L. (1997) Casein kinase II binds to and phosphorylates cytoplasmic dynein. J. Biol. Chem. 272, 5887–5891 doi:10.1074/jbc.272.9.5887 9038206

[35] Vaughan P.S., Leszyk J.D. and Vaughan K.T. (2001) Cytoplasmic dynein intermediate chain phosphorylation regulates binding to dynactin. J. Biol. Chem. 276, 26171–26179 doi:10.1074/jbc.M102649200 11340075

[36] Morgan J.L., Song Y. and Barbar E. (2011) Structural dynamics and multiregion interactions in dynein-dynactin recognition. J. Biol. Chem. 286, 39349–39359 doi:10.1074/jbc.M111.296277 21931160 PMC3234759

[37] Siglin A.E., Sun S., Moore J.K., Tan S., Poenie M., Lear J.D. et al. (2013) Dynein and dynactin leverage their bivalent character to form a high-affinity interaction. PLoS ONE 8, e59453 doi:10.1371/journal.pone.0059453 23577064 PMC3618186

[38] Williams F.J. and Fiedler D. (2015) A fluorescent sensor and gel stain for detection of pyrophosphorylated proteins. ACS Chem. Biol. 10, 1958–1963 doi:10.1021/acschembio.5b00256 26061479

[39] Shears S.B., Gokhale N.A., Wang H. and Zaremba A. (2011) Diphosphoinositol polyphosphates: what are the mechanisms? Adv. Enzyme Regul. 51, 13–25 doi:10.1016/j.advenzreg.2010.09.008 21035493 PMC3507380

[40] Vaughan K.T. and Vallee R.B. (1995) Cytoplasmic dynein binds dynactin through a direct interaction between the intermediate chains and p150^Glued^. J. Cell Biol. 131, 1507–1516 doi:10.1083/jcb.131.6.1507 8522607 PMC2120689

[41] King S.J., Brown C.L., Maier K.C., Quintyne N.J. and Schroer T.A. (2003) Analysis of the dynein-dynactin interaction in vitro and in vivo. Mol. Biol. Cell 14, 5089–5097 doi:10.1091/mbc.E03-01-0025 14565986 PMC284810

[42] Wu M., Dul B.E., Trevisan A.J. and Fiedler D. (2013) Synthesis and characterization of non-hydrolysable diphosphoinositol polyphosphate messengers. Chem. Sci. 4, 405–410 doi:10.1039/C2SC21553E 23378892 PMC3558982

[43] Urnavicius L., Zhang K., Diamant A.G., Motz C., Schlager M.A., Yu M. et al. (2015) The structure of the dynactin complex and its interaction with dynein. Science 347, 1441–1446 doi:10.1126/science.aaa4080 25814576 PMC4413427

[44] Chowdhury S., Ketcham S.A., Schroer T.A. and Lander G.C. (2015) Structural organization of the dynein–dynactin complex bound to microtubules. Nat. Struct. Mol. Biol. 22, 345–347 doi:10.1038/nsmb.2996 25751425 PMC4385409

[45] Barbar E. (2012) Native disorder mediates binding of dynein to NudE and dynactin. Biochem. Soc. Trans. 40, 1009–1013 doi:10.1042/BST20120180 22988856 PMC3785714

[46] Pullikuth A.K., Ozdemir A., Cardenas D., Bailey E., Sherman N.E., Pfister K.K. et al. (2013) Epidermal growth factor stimulates extracellular-signal regulated kinase phosphorylation of a novel site on cytoplasmic dynein intermediate chain 2. Int. J. Mol. Sci. 14, 3595–3620 doi:10.3390/ijms14023595 23434660 PMC3588060

[47] Pfister K.K. (2015) Distinct functional roles of cytoplasmic dynein defined by the intermediate chain isoforms. Exp. Cell Res. 334, 54–60 doi:10.1016/j.yexcr.2014.12.013 25576383 PMC4433767

[48] Yates L.M. and Fiedler D. (2015) Establishing the stability and reversibility of protein pyrophosphorylation with synthetic peptides. ChemBioChem 16, 415–423 doi:10.1002/cbic.201402589 25639821

